# Melange

**Published:** 1885-07

**Authors:** 


### MELANGE.

CHRISTENED.—Dr. F. E. Daniel, of Austin, Texas, sends us the prospectus of "DANIEL'S TEXAS MEDICAL JOURNAL." To those of our readers who were lovers of the editorial writings of the much lamented Gaillard, we commend Dr. Daniel, as the coming "Mephistopheles of Medical Journalism." At least we will venture to so christen the baby.—*Denver Medical Journal.*

PRECAUTIONS AGAINST CHOLERA.—At the request of the Secretary of Treasury, the Secretary of State has appointed inspectors to be attached to all the Cuban Consulates to inspect vessels arriving from infected parts and departing for America. The rapidity with which the disease is spreading in Spain leaves but little room to doubt that it will reach the United States this season, unless the sanitary officers of the Government are unusually active and vigilant. Should America escape a visitation it will certainly be due to the putting in practice of the strictest and most rigid sanitary precautions and the watchfulness of those on the battlements; for the history of the disease (which has been repeated many times) is that when it prevails epidemically in Europe it is always sure to reach America in the course of a year or two. Our people should be on guard and check the first appearance of diarrhœa, and keep everything clean.

The Secretary of Treasury has also instituted a system of marine police to patrol the coast and overhaul all vessels, and ask them when they came and where they go, and all about the condition of the crew, passengers and cargo. If suspected and are inward bound, the boat is sent to quarantine and the Secretary of Treasury notified. This important service is rendered by the Revenue Cutters of the Marine Hospital Service; they are kept going in and out all the time.

Such vigilence, guided and backed by the intelligent officers of the Marine Hospital Service, surely will avail to keep out the dread enemy.

---

TODGERS REDIVIVUS. — An Austin boarding-house madam mulched us *two dollars* for damages, because one of our little "mitherless bairns" dampened the bed one night. Fact! Thus it seems *petit pois* are reckoned amongst the high priced 'extras' by the Austin Todgerses!

